# Hypercapnia, Prognostic Nutritional Index and Length of Stay in Acute Exacerbation of COPD: A Two-Variable Admission Framework

**DOI:** 10.3390/diagnostics16131963

**Published:** 2026-06-24

**Authors:** Orkun Eray Terzi, Nazlı Çetin, Büşra Yıldırım Kafalı, Büşra Çomaklı Özmen, Gülgün Çetintaş Afşar, Seyhan Dülger

**Affiliations:** 1 Department of Pulmonology, Bursa Yüksek İhtisas Training and Research Hospital University of Health Sciences, Bursa 16310, Türkiye; yil.busrayildirim@gmail.com (B.Y.K.); comaklibusra16@gmail.com (B.Ç.Ö.); gulgun.cetintasafsar@sbu.edu.tr (G.Ç.A.); seyhan.dulger@sbu.edu.tr (S.D.); 2 Department of Pulmonology, Afyonkarahisar State Hospital, Afyonkarahisar 03100, Türkiye; nazlicetinbeyaz@gmail.com

**Keywords:** acute exacerbation of COPD, AECOPD, hospital length of stay, hypercapnia, prognostic nutritional index, nutritional–immune status, risk stratification, admission biomarkers

## Abstract

**Background/Objectives:** Established AECOPD prognostic tools (DECAF, BAP-65, PEARL) predict mortality or readmission rather than length of stay (LOS), and no admission-based instrument specifically targets prolonged hospitalization. We tested whether admission PaCO_2_ and the Prognostic Nutritional Index (PNI), reflecting ventilatory failure and nutritional–immune reserve, are independently associated with prolonged LOS and examined their interaction. **Methods:** In this single-center retrospective cohort, 213 adults hospitalized exclusively for AECOPD were analyzed after excluding concomitant pneumonia, pulmonary embolism, decompensated heart failure, and in-hospital deaths. Prolonged hospitalization was pre-specified as LOS > 7 days. Multivariable logistic regression evaluated admission PaCO_2_ (per +10 mmHg) and PNI (per +5 units) with a PaCO_2_ × PNI interaction; continuous LOS was modeled by Gamma regression. Discrimination was compared with DECAF using DeLong’s test. **Results:** Prolonged hospitalization occurred in 83 patients (39.0%). Admission PaCO_2_ was independently associated with prolonged LOS (OR 1.52, 95% CI 1.25–1.88; *p* < 0.001), and PNI showed a borderline association (OR 0.84, 95% CI 0.71–1.00; *p* = 0.049); their interaction was significant but exploratory (OR 1.16, 95% CI 1.02–1.32; *p* = 0.025). In Gamma regression, PaCO_2_ (RR 1.18 per 10 mmHg) and PNI (RR 0.92 per 5 units) remained associated with LOS. The two-variable model achieved an AUC of 0.682, showing discrimination similar to DECAF in this cohort (AUC 0.695; DeLong *p* = 0.76), with optimism-corrected AUC 0.672 and calibration slope 0.96. Within moderate hypercapnia (PaCO_2_ 45–60 mmHg), the prolonged-LOS rate was 44.4% in low-PNI versus 15.6% in high-PNI patients. **Conclusions:** In this single-center retrospective cohort of AECOPD patients surviving to discharge, admission PaCO_2_ and PNI were jointly associated with prolonged hospitalization, reflecting acute ventilatory burden and nutritional–immune reserve. Using only two admission inputs, the framework showed discrimination similar to DECAF without meaningful reclassification gain (IDI −0.02; NRI 0.02). Given only moderate discrimination (AUC ~ 0.68), external validation is required before clinical use, with the main practical value likely in complementary stratification within moderate hypercapnia.

## 1. Introduction

Acute exacerbations of chronic obstructive pulmonary disease (AECOPD) are a major cause of hospitalization and an important contributor to in-hospital cost and mortality. Length of stay (LOS), however, varies widely between patients with similar admission profiles, which makes discharge planning difficult. It also reflects non-clinical factors such as discharge readiness, home oxygen or NIV arrangements, rehabilitation availability, social support, and weekend discharge practices. Established admission-based risk scores (DECAF, BAP-65, and PEARL) were developed to predict short-term mortality or early readmission rather than hospitalization duration [1,2,3]. Models targeting LOS often enroll heterogeneous populations with concomitant pneumonia, pulmonary embolism, or decompensated heart failure, or use in-hospital variables unavailable at admission [4,5]. As a result, an admission-based, AECOPD-specific framework for stratifying prolonged hospitalization is lacking.

Admission arterial PaCO_2_ reflects the severity of acute ventilatory failure and has been associated with adverse short-term outcomes in AECOPD [6]. However, ventilatory failure alone does not explain the in-hospital course; patients with similar blood gases can recover very differently, which points to a role for nutritional and immune status. The Prognostic Nutritional Index (PNI), calculated from serum albumin and absolute lymphocyte count, captures both domains and has been linked to in-hospital outcomes in COPD [7,8]. PaCO_2_ and PNI therefore reflect two different but easily measured aspects of an AECOPD admission: acute ventilatory failure and underlying nutritional–immune reserve. Looking at them together may explain LOS variation better than either alone, and PNI may modify the association between admission hypercapnia and a prolonged stay.

In this single-center retrospective cohort of patients admitted only for AECOPD (excluding concomitant pneumonia, pulmonary embolism, and decompensated heart failure) we tested whether admission PaCO_2_ and PNI are independently associated with prolonged hospitalization (LOS > 7 days). As a pre-specified secondary analysis, we examined their interaction and compared the discrimination of this two-variable admission framework with established AECOPD risk scores. Both hypercapnia and nutritional status have individually been associated with AECOPD outcomes; the contribution of the present study is their combined evaluation for prolonged length of stay rather than the use of either marker alone.

## 2. Methods

### 2.1. Study Design and Ethical Approval

This retrospective, single-center study was conducted at Bursa Yüksek İhtisas Training and Research Hospital, affiliated with the University of Health Sciences (Bursa, Türkiye). The study protocol was approved by the Institutional Clinical Research Ethics Committee (Approval No: 2024-TBEK 2025/12-11; 3 December 2025), and the study was conducted in accordance with the Declaration of Helsinki. The study is reported in accordance with the STROBE statement.

### 2.2. Study Population

Adult patients (≥18 years) hospitalized with a diagnosis of COPD between 1 January 2024 and 31 December 2025 were retrospectively screened through the hospital information system. COPD diagnosis was identified using ICD-10 codes (J44.0, J44.1, J44.9) and confirmed by a documented clinical diagnosis made by a pulmonologist. To improve diagnostic accuracy beyond administrative coding, every record was individually reviewed; inclusion required a physician-documented diagnosis of COPD together with a clinical presentation consistent with an acute exacerbation, and cases in which an alternative acute diagnosis better explained the presentation were excluded according to the criteria detailed below. For patients with multiple admissions during the study period, only the first hospitalization was included. AECOPD was defined as an acute worsening of respiratory symptoms (dyspnea, sputum volume, and/or sputum purulence) in a patient with established COPD, requiring a change in treatment.

To obtain a clinically homogeneous cohort, patients with acute conditions that could independently influence hospitalization duration were excluded. These included asthma or asthma–COPD overlap (documented in the pulmonologist’s note), radiologically confirmed pneumonia with new infiltrates, any other non-COPD acute respiratory pathology, acute pulmonary embolism confirmed by computed tomography pulmonary angiography or ventilation–perfusion scintigraphy, and acute decompensated heart failure confirmed by echocardiography. Patients who died during hospitalization were also excluded (*n* = 12), since early mortality may artificially shorten LOS and bias analyses of prolonged hospitalization. Because in-hospital death and discharge are competing events that truncate length of stay through different mechanisms, retaining fatal cases would conflate two distinct processes; accordingly, the present findings apply to patients who survive to discharge and should not be extrapolated to those at highest risk of in-hospital death.

**Figure 1:**
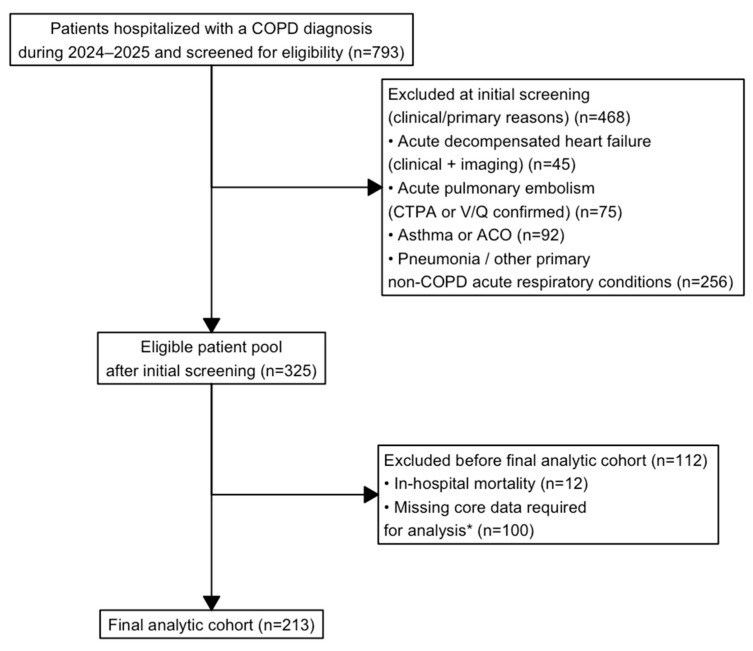
Flowchart of patient screening, exclusions, and final analytic cohort. * Core data required for analysis included arterial blood gas parameters, serum albumin level, and absolute lymphocyte count and patients with missing values in any of these variables were excluded from the final analytic cohort. COPD, chronic obstructive pulmonary disease; ACO, asthma–COPD overlap; CTPA, computed tomography pulmonary angiography; V/Q, ventilation–perfusion scintigraphy.

The sequential application of these exclusion criteria and the resulting analytic cohort are shown in Figure 1.

### 2.3. Data Collection and Variables

All admission data were extracted from the hospital electronic medical record system and defined as the first clinical and laboratory measurements obtained at the time of hospitalization.

Demographic variables included age and sex. Clinical variables included smoking history (ever vs. never smoker), ≥1 exacerbation within the preceding 12 months (based on chart documentation), home non-invasive ventilation (NIV) use, and long-term oxygen therapy (LTOT). Comorbidities were captured from the medical record and analyzed only as a composite binary variable, ‘any comorbidity,’ defined as the presence of at least one of these conditions. In-hospital ventilatory support (noninvasive and invasive mechanical ventilation) and intensive care unit admission were also recorded.

Arterial blood gas parameters at admission (pH, PaCO_2_, and HCO_3_^−^) were recorded; admission PaCO_2_ was the primary variable of interest for ventilatory failure. Laboratory variables included white blood cell, neutrophil, and lymphocyte counts; serum albumin; C-reactive protein (CRP); procalcitonin (PCT); urea; creatinine; and uric acid. PNI was calculated from admission serum albumin and absolute lymphocyte count using the following formula:

PNI = [10 × serum albumin (g/dL)] + [0.005 × absolute lymphocyte count (/mm^3^)].

Serum albumin was measured in g/L; values were converted to g/dL (divided by 10) before calculating the PNI, whereas albumin is reported in g/L in the tables.

The DECAF, BAP-65, and PEARL scores were calculated from admission data according to their original definitions.

### 2.4. Outcomes

The primary outcome was prolonged hospitalization. Length of stay (LOS) was defined as the number of calendar days from hospital admission to discharge. For categorical analyses, prolonged LOS was defined as hospitalization exceeding 7 days. The 7-day threshold was pre-specified in the analysis plan, based on cutoffs reported in prior AECOPD LOS studies and the median LOS observed in Turkish and European cohorts. The median LOS in the present cohort (7 days) matched this a priori choice rather than being used to define it. In secondary analyses, LOS was evaluated as a continuous variable to account for its right-skewed distribution.

### 2.5. Statistical Analysis

All statistical analyses were performed using IBM SPSS Statistics version 29.0 (IBM Corp., Armonk, NY, USA) and R software (version 4.0, R Foundation for Statistical Computing, Vienna, Austria). All tests were two-sided, and a *p*-value < 0.05 was considered statistically significant.

This was a retrospective analysis of all eligible AECOPD admissions during the study period; no a priori sample size calculation was performed because the cohort size was determined by the available case pool. Of 213 included patients, 83 (39.0%) experienced prolonged hospitalization (>7 days). The pre-specified two-variable model included two main predictors (admission PaCO_2_ and PNI) and an exploratory PaCO_2_ × PNI interaction term. With 83 events, this corresponded to approximately 41.5 events per parameter for the main-effects model and approximately 27.7 events per parameter when the interaction term was added—both well above the conventional 10 events-per-variable threshold for logistic regression.

Continuous variables were assessed for normality using the Kolmogorov–Smirnov test together with visual inspection of histograms and Q–Q plots. Normally distributed variables were reported as mean ± standard deviation and non-normally distributed variables as median (minimum–maximum). Categorical variables were expressed as counts and percentages. Group comparisons between patients with and without prolonged LOS were performed using Student’s *t*-test or the Mann–Whitney U test for continuous variables and the chi-square test or Fisher’s exact test for categorical variables, as appropriate.

Three pre-specified model categories were evaluated for prolonged LOS (>7 days): (i) a clinical model including age (per 1-year increase), ≥1 exacerbation in the previous 12 months (yes/no), and any comorbidity (yes/no); (ii) a two-variable model including admission PaCO_2_ (per +10 mmHg) and PNI (per +5 units); and (iii) established clinical risk scores (DECAF, BAP-65, and PEARL), each evaluated as a continuous predictor. As a sensitivity analysis, an expanded clinical model was also evaluated, adding pH, long-term oxygen therapy, home NIV, and smoking status to the clinical model.

The two-variable model was pre-specified and deliberately limited to PaCO_2_ and PNI in order to evaluate their joint contribution while avoiding overfitting; no automated variable selection procedures were applied. Linearity of continuous predictors on the log-odds scale was assessed graphically, and multicollinearity was evaluated using the variance inflation factor (VIF). To explore potential effect modification, a PaCO_2_ × PNI interaction term was tested within the two-variable model as a pre-specified secondary analysis. Throughout, “pre-specified” denotes that the predictors, the 7-day LOS threshold, and the interaction term were defined in the analysis plan before any model was fitted; the study was not based on a separately registered protocol. For the interaction model, PaCO_2_ and PNI were mean-centered (scaled per 10 mmHg and per 5 units, respectively) before forming the product term.

For analyses of LOS as a continuous outcome, a Gamma regression with a log link was used to accommodate the right-skewed distribution. This approach was preferred over log-transformed linear regression because it models skewness directly while preserving interpretability on the original scale. Effects were reported as rate ratios with 95% confidence intervals.

Model performance was evaluated in terms of discrimination, calibration, and internal validation. Discrimination was assessed using the area under the receiver operating characteristic curve (AUC) with 95% confidence intervals; pairwise AUC comparisons were performed using DeLong’s test. Calibration was assessed primarily using calibration plots together with the calibration intercept and slope; the Hosmer–Lemeshow test was reported as a supplementary measure. Internal validation was performed using 1000 bootstrap resamples to obtain optimism-corrected estimates of discrimination (AUC) and calibration slope. Overall model performance was additionally summarized using the Brier score. The incremental predictive value of the two-variable model relative to the clinical and DECAF models was assessed using the continuous net reclassification improvement (NRI) and the integrated discrimination improvement (IDI).

Patients with missing values in any of the core analytic variables (admission PaCO_2_, serum albumin, or absolute lymphocyte count) were excluded prior to model fitting (*n* = 100; Figure 1). Among the 213 included patients, all variables used in the pre-specified models were complete; therefore, complete-case analysis was performed and no imputation procedures were applied.

## 3. Results

A total of 213 patients hospitalized exclusively for AECOPD were included after applying the inclusion and exclusion criteria to 793 screened admissions (Figure 1). The mean age was 69.2 ± 9.1 years and 76.1% were male; age and sex were comparable between patients with and without prolonged hospitalization. A history of ≥1 exacerbation in the previous 12 months was more frequent in the prolonged-LOS group (67.5% vs. 49.2%; *p* = 0.009), and ever smokers were over-represented (94.0% vs. 84.6%; *p* = 0.03). At admission, patients with prolonged LOS had lower arterial pH and higher PaCO_2_ (both *p* < 0.001), whereas HCO_3_^−^ was similar between groups (*p* = 0.80). PNI was lower in the prolonged-LOS group (*p* = 0.005), and absolute lymphocyte count was lower as well (median 1.28 vs. 1.57 × 10^9^/L; *p* = 0.02). Among established clinical scores, DECAF, BAP-65, and PEARL distributions all differed between groups (*p* = 0.03, *p* < 0.001, and *p* = 0.001, respectively). Prolonged hospitalization (>7 days) occurred in 83 patients (39.0%). In-hospital ventilatory support was common: noninvasive ventilation was used in 85 patients (39.9%), invasive mechanical ventilation in 19 (8.9%), and 49 (23.0%) were admitted to intensive care; each was associated with prolonged LOS (all *p* < 0.001). Full baseline characteristics are presented in Table 1.

Multivariable logistic regression results are summarized in Table 2A. In the clinical model, only a history of ≥1 exacerbation in the previous 12 months was independently associated with prolonged LOS (OR 2.22, 95% CI 1.25–4.00; *p* = 0.007), whereas age and any comorbidity were not (Table 2). In the two-variable model, each 10 mmHg increase in admission PaCO_2_ raised the odds of prolonged LOS (OR 1.52, 95% CI 1.25–1.88; *p* < 0.001), and each 5-unit increase in PNI lowered them (OR 0.84, 95% CI 0.71–1.00; *p* = 0.049). The pre-specified PaCO_2_ × PNI interaction was significant (OR 1.16, 95% CI 1.02–1.32; β = 0.147; *p* = 0.025), showing that the deleterious effect of hypercapnia on LOS was amplified in patients with lower nutritional–immune reserve. This interaction is considered exploratory and hypothesis-generating given the modest sample size. In single-predictor models, DECAF (OR 2.16 per point, 95% CI 1.59–3.00; *p* < 0.001), PEARL (OR 1.30, 95% CI 1.11–1.53; *p* = 0.001), and BAP-65 (OR 1.39, 95% CI 1.03–1.89; *p* = 0.03) were each independently associated with prolonged LOS (Table 2).

When LOS was modeled as a continuous outcome with Gamma regression (Table 2B), both predictors were associated with hospitalization duration: each 10 mmHg increase in PaCO_2_ corresponded to an 18% longer expected LOS (RR 1.18, 95% CI 1.11–1.25), and each 5-unit increase in PNI to an 8% shorter expected LOS (RR 0.92, 95% CI 0.88–0.97). Sensitivity analyses across alternative LOS thresholds (>5, >7, and >10 days) showed a consistent direction of effect for PaCO_2_, with effect size increasing as the threshold became more stringent (OR 1.38, 1.52, and 1.63; *p* = 0.003, *p* < 0.001, and *p* < 0.001, respectively). The PNI association was directionally consistent across all thresholds but reached statistical significance only at the primary >7-day cutoff (*p* = 0.049), not at >5 days (*p* = 0.13) or >10 days (*p* = 0.07). Discrimination improved with stricter thresholds (AUC 0.652, 0.682, and 0.731, respectively; Table 3). In a sensitivity analysis excluding the three patients with LOS >30 days, the admission PaCO_2_ association was essentially unchanged (OR 1.50, 95% CI 1.22–1.84; *p* < 0.001), indicating that the findings were not driven by extreme values.

**Figure 2:**
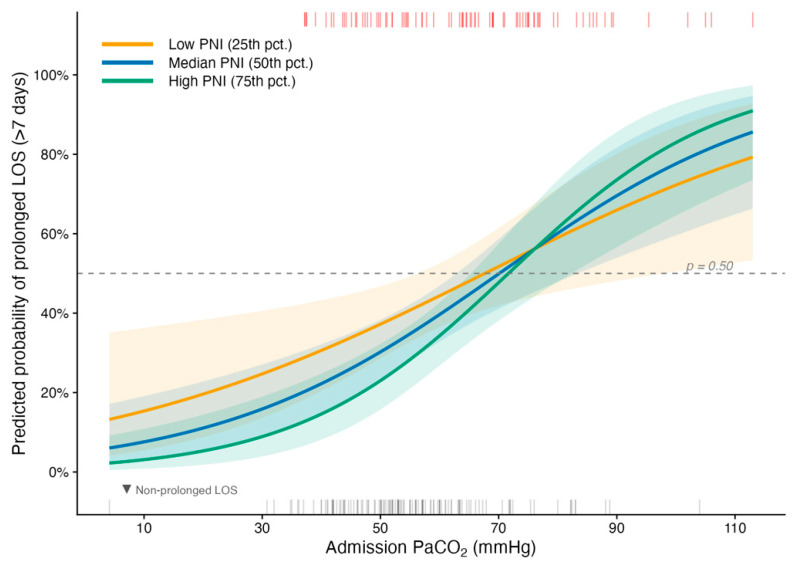
Predicted probability of prolonged hospitalization according to admission PaCO_2_, stratified by prognostic nutritional index (PNI) tertiles. Predicted probabilities of prolonged hospitalization (LOS > 7 days) derived from the multivariable logistic regression model are shown as a function of admission PaCO_2_. Curves are presented for representative values of PNI corresponding to the 25th percentile (low), 50th percentile (intermediate), and 75th percentile (high). Shaded areas indicate 95% pointwise confidence bands. The divergence of curves across the clinically relevant PaCO_2_ range reflects effect modification by PNI, with attenuation at extreme PaCO_2_ levels. This effect modification is exploratory and hypothesis-generating and requires external validation.

The predicted probability of prolonged hospitalization across admission PaCO_2_, stratified by PNI tertile, is shown in Figure 2. Predicted probabilities diverged most across PNI strata in the mid-range of PaCO_2_ (approximately 45–60 mmHg) and converged at the extremes. For clinical interpretation, patients were grouped by PaCO_2_ category (≤45, 45–60, and >60 mmHg) and PNI tertile (low ≤ 41, intermediate 41–47, high > 47); the joint risk stratification is shown in Table 4. The observed prolonged-LOS rate ranged from 15.6% in the moderate-PaCO_2_/high-PNI cell to 69.7% in the high-PaCO_2_/low-PNI cell. Within the moderate PaCO_2_ range, the rate was 44.4% in low-PNI patients versus 15.6% in high-PNI patients.

**Figure 3:**
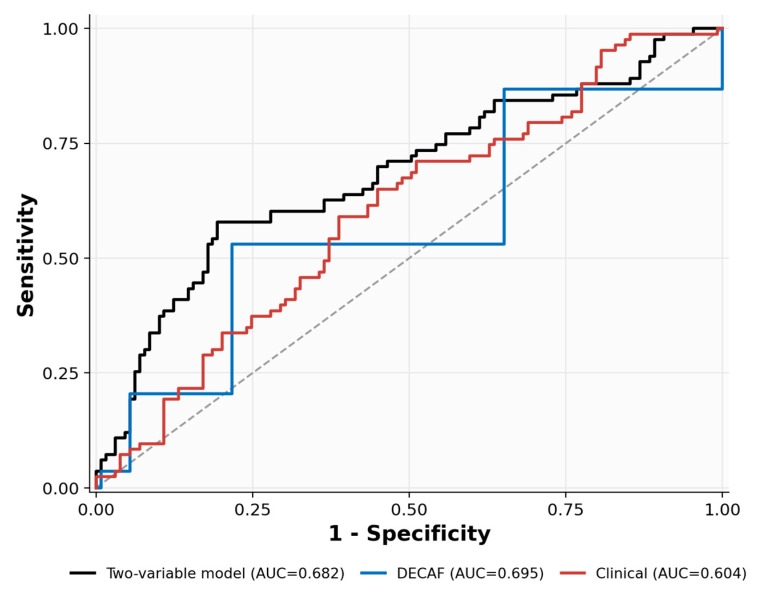
Receiver operating characteristic curves of admission-based models for predicting prolonged hospital stay (>7 days) in acute exacerbation of COPD. ROC curves comparing the two-variable model (PaCO_2_ + PNI), DECAF, and clinical models.

**Figure 4:**
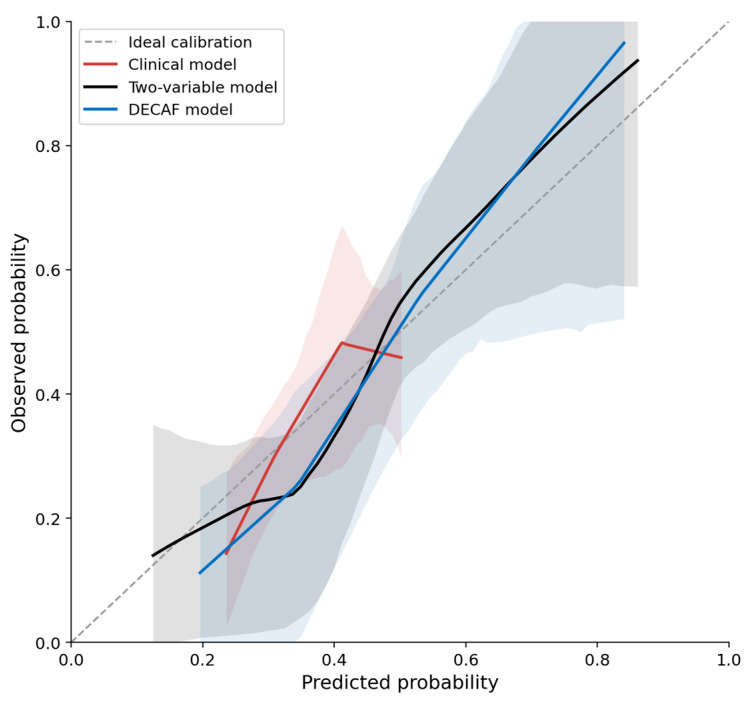
Bootstrap-corrected calibration of the two-variable model for prolonged hospital stay. Shaded areas represent 95% confidence intervals around the calibration curves.

Model discrimination and calibration are summarized in Table 5. The two-variable model had an AUC of 0.682 (95% CI 0.606–0.759), comparable to the DECAF model (AUC 0.695, 95% CI 0.625–0.764; DeLong’s test, *p* = 0.76). The clinical model had lower discrimination (AUC 0.604), and BAP-65 (AUC 0.585) and PEARL (AUC 0.629) also showed weaker discrimination than DECAF and the two-variable model. In pairwise DeLong comparisons against the two-variable model, discrimination did not differ significantly from DECAF (*p* = 0.76) or PEARL (*p* = 0.28), whereas the two-variable model showed significantly higher discrimination than BAP-65 (*p* = 0.045). An expanded clinical model that also included pH, LTOT, home NIV, and smoking status reached an AUC of 0.706, comparable to the two-variable model (0.682) and DECAF (0.695) but using more inputs; only lower pH (*p* = 0.001) and smoking status (OR 3.47, 95% CI 1.13–10.67; *p* = 0.03) were independently associated with prolonged LOS. Relative to the clinical model, the two-variable model improved prediction (IDI = 0.068; continuous NRI = 0.214). Relative to DECAF, the two-variable model provided no meaningful incremental performance (IDI = −0.016; continuous NRI = 0.022), consistent with their comparable AUCs. On 1000 bootstrap resamples, the optimism-corrected AUC for the two-variable model was 0.672 (apparent 0.682; optimism 0.011), and the optimism-corrected calibration slope was 0.96, indicating minimal overfitting. ROC curves are shown in Figure 3 and bootstrap-corrected calibration in Figure 4. These results characterize associations, the apparent discrimination, and the internal validity of admission-based models; they do not constitute external validation of a clinical prediction tool.

## 4. Discussion

Established prognostic instruments for AECOPD (DECAF, PEARL, and BAP-65) were developed to predict short-term mortality or early readmission and do not directly target prolonged hospitalization, an endpoint relevant to discharge planning and bed management [1,2,3]. In this single-center retrospective cohort, restricted to AECOPD admissions without concomitant acute conditions that independently prolong hospitalization, admission arterial PaCO_2_ and the PNI were each associated with prolonged length of stay, and the two-variable model (PaCO_2_ + PNI) achieved discrimination comparable to DECAF (AUC 0.682 vs. 0.695; DeLong’s test, *p* = 0.76), indicating similar but not equivalent discrimination. Recovery duration after an AECOPD episode therefore appears to be associated with both the severity of acute ventilatory failure and underlying nutritional–immune reserve, two dimensions not directly captured by existing mortality- or readmission-oriented scores.

Hypercapnia at admission is a direct biochemical marker of acute ventilatory insufficiency in AECOPD. Resolving hypercapnic respiratory failure (restoring ventilation, correcting acidosis, and weaning from ventilatory support) requires a recovery effort that increases with the severity of the exacerbation [6]. Inspiratory muscle dysfunction itself contributes to and predicts hypercapnic failure in COPD [9], creating a bidirectional relationship between ventilatory mechanics and CO_2_ retention; recovery therefore depends both on resolving the acute insult and on restoring inspiratory muscle function. Prior studies in unselected AECOPD cohorts have identified admission blood gas abnormalities among determinants of extended hospital stay [4,5,10,11], and analyses focused on hypercapnic respiratory failure confirm the independent contribution of PaCO_2_ to hospitalization duration [12,13]. In the present cohort, each 10-mmHg increase in admission PaCO_2_ was independently associated with 52% higher odds of prolonged LOS and an 18% longer expected stay in Gamma regression. This association persisted across both analytical frameworks (logistic and Gamma) and across three sensitivity thresholds, with a graded increase in effect size at stricter cutoffs (OR 1.38, 1.52, and 1.63 for >5, >7, and >10 days, respectively). The absence of between-group differences in admission HCO_3_^−^ is consistent with an acute rather than chronic component of hypercapnia, although a chronic compensatory contribution cannot be fully excluded from a single admission measurement.

The PNI, derived from serum albumin and absolute lymphocyte count, reflects a different aspect of host biology than acute-phase markers such as CRP: whereas CRP reflects the intensity of the acute insult, PNI reflects the reserve available to recover from it. Serum albumin reflects nutritional reserve and hepatic synthetic capacity, both reduced by chronic pulmonary disease and acute systemic inflammation. In COPD, hypoalbuminemia frequently coexists with sarcopenia, which reduces respiratory muscle reserve and limits the ability to sustain the increased ventilatory work of an exacerbation [14]. Lymphocyte count reflects immune competence for pathogen clearance and inflammation resolution. Lower PNI has been associated with adverse in-hospital outcomes and higher short-term mortality in AECOPD [7,15,16] and in critically ill populations [8,17]. In a recent retrospective cohort of 839 elderly AECOPD patients, PNI-defined malnutrition was associated with prolonged hospitalization (>7 days; OR 1.88, 95% CI 1.43–2.47) [16]; the present analysis extends those findings by modeling PNI as a continuous predictor and integrating it with admission PaCO_2_. The PNI association reached borderline significance in logistic regression (*p* = 0.049) but was consistent in Gamma regression (an 8% shorter expected LOS per 5-unit increase), supporting a robust association across distinct distributional assumptions rather than a chance finding. This fits a broader pattern in which reduced physiological reserve (nutritional, as captured by PNI, or physical, as captured by frailty) predicts adverse outcomes in COPD; admission-graded frailty has recently been linked to major adverse cardiovascular events in this population [18].

The pre-specified PaCO_2_ × PNI interaction was statistically significant (OR 1.16, 95% CI 1.02–1.32; β = 0.147; *p* = 0.025), with a stronger PaCO_2_–LOS association at lower PNI values. This pattern is mechanistically plausible: the metabolic and immunological demands of recovering from hypercapnic respiratory failure (sustained respiratory muscle work, acid–base correction, resolution of systemic inflammation) are increased when nutritional and immunological reserve is reduced. The predicted-probability curves in Figure 2 illustrate this graphically: divergence across PNI strata is greatest in the moderate hypercapnia range (PaCO_2_ 45–60 mmHg) and converges at extreme values, consistent with a ceiling effect—at very high PaCO_2_, prolonged stay becomes likely regardless of PNI. The inclusion of the interaction term did not improve model discrimination, indicating an interpretive rather than a classification gain.

No admission-based instrument has been specifically developed to predict prolonged hospitalization in AECOPD. DECAF was selected as the reference comparator because it is the most widely used admission-based multi-component score, although it was developed and externally validated as a mortality predictor [1,19] and has been used to guide discharge in low-risk patients [20]. The two-variable model achieved an AUC of 0.682, comparable to DECAF (AUC 0.695; DeLong’s test, *p* = 0.76), using only admission PaCO_2_ from routine arterial blood gas and PNI from standard biochemistry and differential count. This level of discrimination is below the thresholds typically required for standalone clinical decision support and is not intended for isolated use; rather, the two-variable framework is intended as a complementary layer that addresses an endpoint (anticipated recovery duration) not directly targeted by existing scores. Relative to the clinical model, the two-variable model improved prediction (IDI = 0.068; continuous NRI = 0.214) [21,22]. Relative to DECAF, the two-variable model provided no meaningful incremental performance (IDI = −0.016; continuous NRI = 0.022) but reached comparable discrimination using only two routinely measured variables instead of five. Internal validation confirmed minimal optimism (optimism-corrected AUC 0.672; calibration slope 0.96), although this within-cohort bootstrap correction does not replace external validation in independent populations. Within the moderate PaCO_2_ range (45–60 mmHg), the joint risk grid (Table 4) showed a nearly threefold difference in prolonged-LOS rate between low- and high-PNI strata (44.4% vs. 15.6%), suggesting that PNI provides the largest practical contribution where ventilatory burden alone is indeterminate. An expanded clinical model incorporating pH, LTOT, home NIV, and smoking reached comparable discrimination (AUC 0.706) but required more inputs; because pH and PaCO_2_ both reflect respiratory acidosis and are collinear, pH was not added to the two-variable model, supporting the efficiency of the two-variable approach.

Strengths of the analysis include systematic exclusion of acute comorbid conditions that independently prolong hospitalization, pre-specification of both predictors and the interaction term, consistent results across logistic and Gamma regression frameworks compared against DECAF, and stability of the PaCO_2_ association across three alternative LOS thresholds. Several limitations should nevertheless be considered. The retrospective single-center design and modest sample size limit generalizability, and the model has not been externally validated; performance in cohorts with different demographic, spirometric, or institutional characteristics is unknown. The 7-day threshold corresponds to the cohort median and aligns with prior AECOPD LOS studies but is to some extent arbitrary; classification performance may differ under alternative cutoffs, although sensitivity analyses across >5, >7, and >10 days showed directional stability. An AUC of approximately 0.68 is appropriate for a two-variable exploratory framework but insufficient for standalone clinical use. Spirometric data, explicit GOLD stage, and symptom scores (mMRC/CAT) were not available; residual confounding by COPD severity, symptom burden, or conditions associated with chronic hypercapnia (e.g., obesity hypoventilation syndrome) cannot be excluded, although the absence of between-group differences in admission HCO_3_^−^ partially mitigates this concern. A single admission HCO_3_^−^ measurement nevertheless cannot fully separate acute from chronic compensatory contributions. Body mass index and detailed pack-year data were not available, which limits adjustment for body composition and cumulative tobacco exposure—both relevant to nutritional and ventilatory status. In-hospital ventilatory support was recorded and is reported in the Results; however, NIV duration and a formal definition of NIV failure were not separately captured, and escalation to invasive ventilation or intensive care was used as a proxy for treatment failure. Because these are in-hospital treatments on the causal pathway between admission hypercapnia and LOS, they were deliberately excluded from the admission-based two-variable model rather than treated as independent confounders. Hospitalization duration is also influenced by non-clinical factors (discharge readiness, social support, institutional bed management) not measured here. In-hospital deaths were excluded because death truncates LOS in a way mechanistically distinct from discharge, which restricts applicability to surviving patients. Baseline characteristics of these 12 patients were not abstracted in this retrospective dataset; with only 12 fatal cases, a formal comparison with the analytic cohort would in any case be statistically underpowered and potentially misleading. In addition, 100 of 325 eligible patients were excluded for missing core data and could not be compared with the analytic cohort; because admission arterial blood gas is the core variable most likely to be missing (it is obtained primarily when ventilatory failure is suspected), the cohort may be enriched for more severe or hypercapnic presentations, which limits generalizability to milder exacerbations. The PaCO_2_ × PNI interaction warrants additional caution. With 213 patients and 83 prolonged-stay events, the confidence interval around the interaction coefficient is wide, and a single *p*-value of 0.025 should be regarded as preliminary evidence of effect modification rather than a confirmed phenomenon. Because the interaction was pre-specified rather than identified through multiple testing, additional correction was not applied; however, this does not eliminate the need for independent replication. Prospective studies with a priori interaction testing and adequate statistical power are required before this effect modification can be considered established or used to guide clinical decisions.

## 5. Conclusions

Admission PaCO_2_ and the Prognostic Nutritional Index are associated with prolonged hospitalization in AECOPD, reflecting acute ventilatory burden and nutritional–immune reserve. When considered together, they provide a parsimonious admission-based framework for early risk assessment using routinely available data, with discrimination similar to DECAF (AUC ~ 0.68). This level of discrimination is insufficient for standalone clinical decision-making; until external validation in larger prospective cohorts is available, PNI may be most useful as a complementary signal alongside DECAF in patients with moderate hypercapnia, where ventilatory burden alone is indeterminate. Because in-hospital deaths were excluded, these findings apply to patients who survive to discharge.

## Figures and Tables

**Table 1:** Baseline characteristics of patients with acute exacerbation of COPD stratified by length of hospital stay.

	Overall *n* = 213	LOS ≤ 7 (Group 1) *n* = 130	LOS > 7 (Group 2) *n* = 83	*p*
Age	69.23 ± 9.08	69.23 ± 9.09	69.10 ± 9.14	0.99
Male sex, *n* (%)	162 (76.1)	94 (72.3)	68 (81.9)	0.11
Ever smoking history, *n* (%)	188 (88.3)	110 (84.6)	78 (94)	0.03
At least one exacerbation in the previous 12 months, *n* (%)	120 (56.3)	64 (49.2)	56 (67.5)	0.009
Any comorbidity, *n* (%)	152 (71.4)	90 (69.2)	62 (71.4)	0.39
Long-term oxygen therapy, *n* (%)	67 (31.5)	35 (26.9)	32 (38.6)	0.08
Home non-invasive ventilation, *n* (%)	24 (11.3)	11 (8.5)	13 (15.7)	0.11
Arterial pH at admission	7.36 (7.06–7.53)	7.37 (7.09–7.53)	7.34 (7.06–7.47)	<0.001
PaCO_2_ at admission, mmHg	56.20 (32–113)	53.50 (32–104)	64.50 (37–113)	<0.001
HCO_3_^−^ at admission, mmol/L	27.50 (15.20–51.50)	27 (17.50–51.50)	27.70 (15.20–39.60)	0.80
White blood cell count (WBC), ×10^9^/L	10.63 (2.56–26.64)	11.20 (2.56–29.90)	10.53 (5.01–26.64)	0.38
Neutrophil count, ×10^9^/L	7.80 (1.93–23.64)	7.92 (1.93–23.04)	7.79 (0.60–23.64)	0.68
Lymphocyte count, ×10^9^/L	1.44 (0.19–9.31)	1.57 (0.19–7.21)	1.28 (0.20–9.31)	0.02
Albumin, g/L	35.46 ± 5.29	36.20 ± 5.13	34.80 ± 5.07	0.08
Urea, mg/dL	20 (5–58)	21 (5–50)	19.2 (9–58)	0.82
Creatinine, mg/dL	0.83 (0.29–5.96)	0.85 (0.29–2.68)	0.80 (0.48–5.96)	0.87
C-reactive protein (CRP), mg/L	29 (0.72–339)	28.7 (1.57–274.65)	24.60 (0.72–339)	0.24
Procalcitonin (PCT), ng/mL	0.045 (0–27.05)	0.040 (0–14.83)	0.050 (0–27.05)	0.64
Uric acid, mg/dL	5.2 (1.01–13)	5.2 (2–13)	5.15 (1.01–11.60)	0.63
Prognostic Nutritional Index (PNI)	43.40 (24.80–82.95)	44.07 (24.80–75.45)	41.25 (26.90–82.95)	0.005
Length of Stay, day	7 (1–68)	5 (1–7)	11 (8–68)	<0.001
DECAF score	1 (0–4)	1 (0–4)	1 (0–4)	0.03
BAP-65 score	1 (0–4)	1 (0–4)	1 (0–4)	<0.001
PEARL score	2 (0–7)	2 (0–7)	3 (1–7)	0.001

Values are presented as mean ± standard deviation for normally distributed variables and as median (minimum–maximum) for non-normally distributed variables. Categorical variables are presented as *n* (%).

**Table 2:** Multivariable regression analyses evaluating admission-based predictors of prolonged and continuous hospital stay in acute exacerbation of COPD. (**A**) Logistic regression models. (**B**) Gamma regression analysis for continuous LOS.

**(A)**					
**Predictor**	**Clinical OR (95% CI),** ***p***	**Two-Variable OR (95% CI),** ***p***	**DECAF OR (95% CI),** ***p***	**PEARL OR (95% CI),** ***p***	**BAP-65 OR (95% CI),** ***p***
≥1 exacerbation (yes)	2.22 (1.25–4.00) 0.007	—	—	—	—
Age (per 1 year)	1.00 (0.97–1.03) 0.93	—	—	—	—
Any comorbidity	1.38 (0.73–2.65) 0.33	—	—	—	—
PaCO_2_ (per +10 mmHg)	—	1.52 (1.25–1.88) <0.001	—	—	—
PNI (per +5 points)	—	0.84 (0.71–1.00) 0.049	—	—	—
PaCO_2_ × PNI (interaction)		β = 0.147; *p* = 0.025			
DECAF (per 1 point)	—	—	2.16 (1.59–3.00) <0.001	—	—
PEARL (per 1 point)	—	—	—	1.30 (1.11–1.53) 0.001	—
BAP-65 (per 1 point)	—	—	—	—	1.39 (1.03–1.89) 0.03
**(B)**					
**Predictor**		**RR (95% CI)**		***p***	
PaCO_2_ (per +10 mmHg)		1.18 (1.11–1.25)		<0.001	
PNI (per +5 points)		0.92 (0.88–0.97)		0.003	

The interaction term represents the product of mean-centered PaCO_2_ (per 10 mmHg) and PNI (per 5 units). (**A**) presents multivariable logistic regression models for prolonged hospital stay (LOS > 7 days). (**B**) presents gamma regression analysis (log-link) for continuous LOS. Odds ratios (OR) and rate ratios (RR) are presented with 95% confidence intervals. The interaction term represents the product of mean-centered PaCO_2_ (per 10 mmHg) and PNI (per 5 units).

**Table 3:** Sensitivity analysis across alternative LOS thresholds.

Threshold	Events, *n*	PaCO_2_ OR (95% CI)	*p*-Value	PNI OR (95% CI)	*p*-Value	AUC (95% CI)
>5 days	141	1.38 (1.12–1.70)	0.003	0.88 (0.75–1.04)	0.13	0.652 (0.577–0.727)
>7 days (primary)	83	1.52 (1.25–1.88)	<0.001	0.84 (0.71–1.00)	0.049	0.682 (0.607–0.759)
>10 days	50	1.63 (1.31–2.04)	<0.001	0.83 (0.68–1.02)	0.07	0.731 (0.648–0.814)

Odds ratios are expressed per 10 mmHg increase in PaCO_2_ and per 5-unit increase in PNI. Models include both predictors entered simultaneously without an interaction term. AUC, area under the receiver operating characteristic curve.

**Table 4:** Joint risk stratification by admission PaCO_2_ categories and PNI tertiles.

PaCO_2_ Category	Low PNI (≤41)	Intermediate PNI (41–47)	High PNI (>47)	Total
≤45 mmHg	3/12 (25.0%)	4/16 (25.0%)	4/13 (30.8%)	11/41 (26.8%)
45–60 mmHg	12/27 (44.4%)	7/29 (24.1%)	5/32 (15.6%)	24/88 (27.3%)
>60 mmHg	23/33 (69.7%)	13/25 (52.0%)	12/26 (46.2%)	48/84 (57.1%)
Total	38/72 (52.8%)	24/70 (34.3%)	21/71 (29.6%)	83/213 (39.0%)

Values are presented as number of events/total number of patients within each subgroup (%), where events correspond to prolonged hospitalization (LOS > 7 days). PaCO_2_ categories used left-closed intervals (≤45, >45 to ≤60, and >60 mmHg); PNI tertiles were low (≤41), intermediate (>41 to ≤47), and high (>47), so that patients at cutoff values are assigned unambiguously to a single category.

**Table 5:** Discrimination, calibration, and internal validation performance of admission-based models for predicting prolonged hospital stay.

Model	Apparent AUC (95% CI)	Optimism (Bootstrap, 1000 Resamples)	Optimism-Corrected AUC	Brier Score	Optimism-Corrected Calibration Slope
Clinical Model	0.604 (0.527–0.681)	0.032	0.573	0.230	0.80
Two-variable model	0.682 (0.606–0.759)	0.010	0.672	0.213	0.96
DECAF	0.695 (0.625–0.764)	0.001	0.694	0.209	1.04
PEARL	0.629 (0.554–0.705)	0.004	0.625	0.226	1.12
BAP-65	0.585 (0.511–0.660)	0.005	0.580	0.233	1.32

Apparent AUC is the area under the ROC curve from the model fitted on the full cohort. Optimism (mean over 1000 bootstrap resamples) was subtracted to yield the optimism-corrected AUC. The optimism-corrected calibration slope reflects shrinkage on internal validation; values close to 1 indicate well-calibrated predictions. Brier score measures overall predictive accuracy (lower is better).

## Data Availability

The data presented in this study are available on reasonable request from the corresponding author. The data are not publicly available due to ethical restrictions.
